# Search and comparison of (epi)genomic feature patterns in multiple genome browser tracks

**DOI:** 10.1186/s12859-020-03781-2

**Published:** 2020-10-19

**Authors:** Arnaud Ceol, Piero Montanari, Ilaria Bartolini, Stefano Ceri, Paolo Ciaccia, Marco Patella, Marco Masseroli

**Affiliations:** 1 Center for Genomic Science of IIT@SEMM, Fondazione Istituto Italiano di Tecnologia (IIT), 20139 Milan, Italy; 2 IEO, European Institute of Oncology IRCCS, 20141 Milan, Italy; 3 DISI, Università di Bologna, 40136 Bologna, Italy; 4 Dipartimento di Elettronica, Informazione e Bioingegneria, Politecnico di Milano, 20133 Milan, Italy

**Keywords:** Computational genomics, Genome-wide pattern-search, Visual analytics, Genome browser

## Abstract

**Background:**

Genome browsers are widely used for locating interesting genomic regions, but their interactive use is obviously limited to inspecting short genomic portions. An ideal interaction is to provide patterns of regions on the browser, and then extract other genomic regions over the whole genome where such patterns occur, ranked by similarity.

**Results:**

We developed SimSearch, an optimized pattern-search method and an open source plugin for the Integrated Genome Browser (IGB), to find genomic region sets that are similar to a given region pattern. It provides efficient visual genome-wide analytics computation in large datasets; the plugin supports intuitive user interactions for selecting an interesting pattern on IGB tracks and visualizing the computed occurrences of similar patterns along the entire genome. SimSearch also includes functions for the annotation and enrichment of results, and is enhanced with a Quickload repository including numerous epigenomic feature datasets from ENCODE and Roadmap Epigenomics. The paper also includes some use cases to show multiple genome-wide analyses of biological interest, which can be easily performed by taking advantage of the presented approach.

**Conclusions:**

The novel SimSearch method provides innovative support for effective genome-wide pattern search and visualization; its relevance and practical usefulness is demonstrated through a number of significant use cases of biological interest. The SimSearch IGB plugin, documentation, and code are freely available at https://deib-geco.github.io/simsearch-app/ and https://github.com/DEIB-GECO/simsearch-app/.

## Background

Next Generation Sequencing (NGS) technologies allow the production of high amounts of reliable high-throughput datasets about a variety of biomolecular signals in a single study [1]; such data are usually collected in huge repositories by large research consortia, such as the Encyclopedia of DNA Elements (ENCODE) [2] or the Roadmap Epigenomics [3]. The processing of such signals permits the extraction of multiple genomic and epigenomic features, whose comprehensive evaluation enables the functional characterization of (epi)genomic regions. In particular, the characterization of chromatin states using combinations of chromatin modification, open chromatin readout, and/or transcription factor binding patterns has been shown as a powerful approach to discover regulatory regions and their cell type specific activity patterns, and to interpret disease-association studies [4–6].

Two main computational approaches and tools have been proposed for this characterization:

1. *ChromHMM* [7], an automated computational system for learning chromatin states, is based on a multivariate Hidden Markov Model [8] that models the observed combination of chromatin marks using a product of independent Bernoulli random variables [4]; it enables robust learning of complex patterns of many chromatin modifications.
2. *Segway* [9, 10] uses a dynamic Bayesian network method [11, 12] that identifies joint patterns of histone modification, transcription factor and open chromatin readouts from multiple NGS functional genomics experiments.

Such systems typically operate on big NGS datasets of epigenomic signals using complex statistical approaches and require powerful computational infrastructures to keep their processing time sufficiently low. Together with the segmentation in multiple different states of the considered (epi)genome, they also provide the identified epigenomic feature patterns that characterize the segmented genomic regions. Such patterns have now been extracted for several different normal and pathological conditions and are available in public repositories [4, 5, 13]; this makes conceptually possible to directly use them for the identification of specific regulatory regions in newly sequenced genomes.

Several genome browsers are available, either as a Web application (e.g., *UCSC Genome Browser* [14], *JBrowse* [15]), or as a desktop application (e.g., *Integrative Genomics Viewer (IGV)* [16], *Integrated Genome Browser (IGB)* [17, 18]). These tools allow the user to visually examine and identify interesting patterns within multiple tracks, i.e., (epi)genomic regions that are at a given distance from one another in different tracks of the genome browser; such patterns might, for example, describe DNA areas that regulate gene expressions, including heterogeneous (epi)genomic features (e.g., histone modification and/or different transcription factor binding enriched regions). Discovering such patterns along the whole genome is very important for biologically interpreting NGS experimental results and comprehending biomolecular phenomena; however, once such patterns are identified in the browser, searching the entire genome for their occurrences is a task with a high computational complexity, and it is thus currently not directly supported in genome browsers.

In a previous paper [19], we demonstrated that it is algorithmically possible to search efficiently genome-wide for even complex multi-region patterns on multiple (epi)genomic tracks. Leveraging on those results, we developed an efficient algorithm for pattern searching, so as to quickly find genomic regions similar to a given pattern, within a large collection of (epi)genomic data. In this paper, we describe and discuss the substantial extension of such algorithm in support of a variety of genomic visual analytics tasks. Towards this goal, we implemented the new pattern-search method within SimSearch, an open source IGB plugin supporting an intuitive interaction with the user, allowing her to both visually select an interesting pattern on the loaded IGB tracks, or within predefined patterns, and visualize occurrences of similar patterns found within the entire genome. We enhanced the developed IGB plugin by creating an associated Quickload repository with several epigenomic feature datasets from ENCODE and Roadmap Epigenomics, so as to be easy and efficient to use for pattern-search. Finally, we also enriched SimSearch with a set of additional functions for the annotation and functional enrichment of results, to ease their biological interpretation on the related (epi)genomic context. We believe that the results of this work and the publicly available SimSearch IGB plugin are of broad interest and relevance to a wide community of scientists and bioinformaticians.

## Implementation

### Pattern-search algorithm

We informally define a “pattern” as a collection of regions, each with a number of attributes of various types, that are present on one or more tracks, where a track is a sequence of (epi)genomic regions increasingly ordered according to their genomic coordinates.

Pattern matching is typically solved through a cost-based approach, where lower cost implies higher similarity and minimum cost identifies best matching. Yet, the pattern matching problem for the search of such a defined pattern, genome-wide on a set of target tracks, is hard, even when each target track is *a priori* matched to a single corresponding pattern track and the total number *M* of regions in the pattern is much lower than the total number *N* of regions in the target tracks. However, in [19] we demonstrated that the complexity of such search can be drastically reduced to the order of $O(MN^2)$; this can be obtained by adopting a “root”-element approach (i.e., fixing the matching between an element/region of the pattern and a region of the corresponding target track, for each pattern region alternatively) and a dynamic programming algorithm (i.e., recursively decomposing the whole search in sub-searches, each one for the sub-set of pattern regions remaining after fixing the previous root-region, for each (sub)pattern root-region matched). Furthermore, given the properties of the (epi)genomic region tracks (for which sequences of regions are increasingly ordered and $M \ll N$) and considering that the optimal matching of a pattern region has to be a “close” region in the corresponding target track, we showed that it is possible to obtain the best matching for each region of the pattern in the target tracks through a windowed binary search. The resulting windowed dynamic programming algorithm can make the complexity drop down to $O(N \log (N))$, making it applicable also to (very) large datasets.

Based on the results in [19], we developed an optimized pattern-search algorithm able to efficiently discover genomic region sets that are similar to a given pattern within a large set of (epi)genomic data. In particular, to search for a pattern in a “target” track *T* that is similar to a “query” pattern *Q*, we follow a cost-based approach, where lower cost implies high similarity. We defined a cost function $C_f(Q,T)$, expressing the cost of a matching *f* between *Q* and *T*, as the (squared) sum of distances between query pattern elements $Q_i$ and associated target track regions $T_{f(i)}$ relative to the first (root) matching pair: $C_f(Q,T) = \sum _i \left( T_{f(i)} - Q_i - \left( T_{f(1)} - Q_1 \right) \right) ^2$. The final goal is to find the matching with minimum cost. The considered model represents each region as a single point, corresponding to the region center. If region lengths are deemed to be relevant for the specific problem at hand, they can be modeled as a region attribute. In such a case, the cost function $C_f(Q,T)$ also includes a measure of (dis-)similarity between the features (attributes) of pattern and target track regions; thus, also region attribute values (and their length) are considered in the developed pattern-search algorithm. Furthermore, in the pattern definition, each pattern track can be defined as a *perfect matching* track (when it includes regions that *must* be present in the results), *partial matching track* (when it includes regions that *might* be missing in the results; candidate patterns whose regions remain unmatched have higher cost), or *negative matching track* (when it includes regions that *should not* appear in the solution; candidate patterns enclosing these regions are removed from the solution search space).

**Fig. 1:**
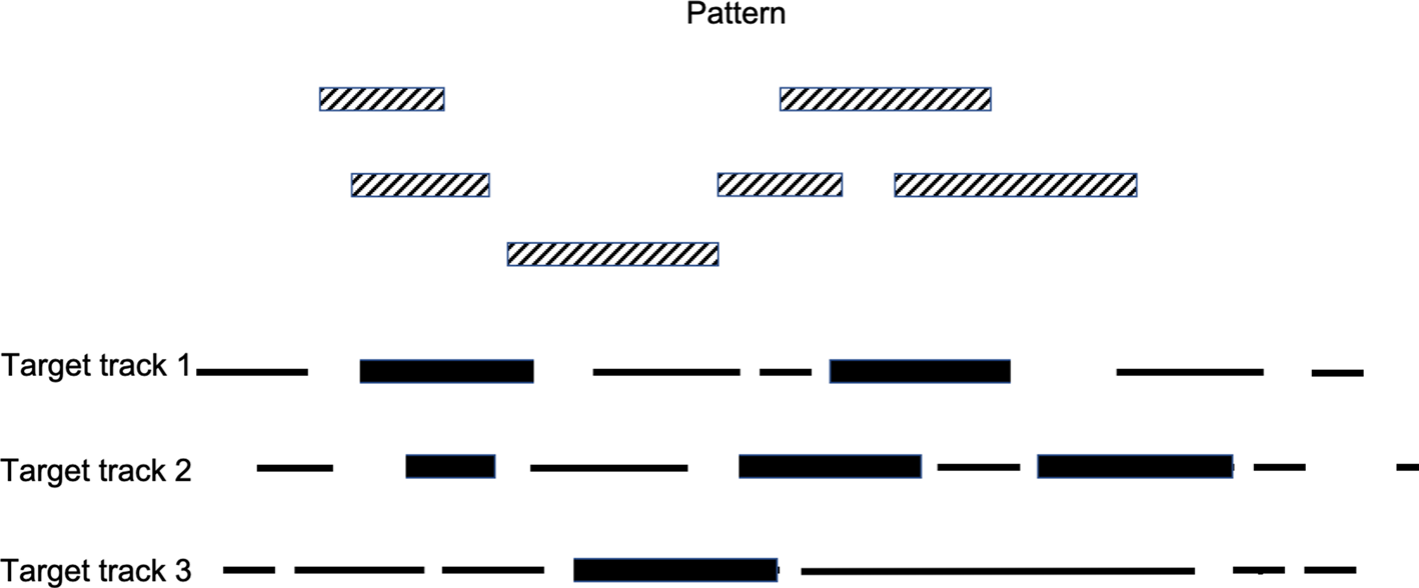
Example of pattern-search result on target tracks, with high structural similarity to the searched pattern: Inter-region distances of the pattern are (approximately) preserved in the regions of the result, both along the same target track and across different tracks

Each result of our pattern-search algorithm is a collection of regions with properties similar to the searched pattern, where similarity applies both to their spatial configuration (structural similarity) and to their feature values (region attribute similarity). As Fig. 1 shows, structural similarity ignores absolute coordinate values of regions, focusing on inter-region distances.

### IGB plugin

IGB is an open source visual analytics platform with high capability to consume data from diverse sources and easily extendable with plugins [18]; we took advantage of its features and Apps API to integrate our developed pattern-search method within IGB and make it of practical utility for genomic visual analytics. We did so by implementing our pattern-search algorithm within a new, open source IGB plugin/App, named SimSearch, compatible with the current IGB version (9.1.4), using Java 8 programming language and Genoviz Software Development Kit [20]. The SimSearch App, its full documentation, and a video showing its step-by-step use are freely available at https://deib-geco.github.io/simsearch-app/, while its code can be found at https://github.com/DEIB-GECO/simsearch-app/.

Genomes and genomic annotations are managed directly by IGB, which provides access to most relevant genomes and is compatible with most common file formats (like, for example, BED, bigBed, VCF, GTF, etc.). IGB also provides integrated access to several datasets, including those offered by the Distributed Annotation System (DAS) server of the University of California at Santa Cruz (UCSC) and the data from the ENCODE project. In addition, IGB can also communicate with a data sharing structure named Quickload [18].

In order to help the reader to reproduce our results or run new analyses without having to deal with the preparation of new files, we created a Quickload server where we stored all the data collected and pre-processed for their use described in this paper (see Supplementary Material section S1). It contains peak regions from ChIP-seq experiments for histone marks and transcription factors from several cell lines from ENCODE [2] and Roadmap Epigenomics [13] projects, as well as epigenomic annotations from ChromHMM and Segway, and processed contact maps from [21] (see Supplementary Material section S1).

### Input data

Our SimSearch IGB App works on any track that includes discrete regions (such as those from BED, bigBed, VCF, or GTF file formats); it is yet unable to manage continuous regions, like those stored in WIG or bigWig files, since this would require a different representation of regions in the pattern-search algorithm (see previous * Pattern-search algorithm section)*. Furthermore, SimSearch directly manages transcription start site (TSS) annotations, which can be automatically extracted from the IGB genome data as a pattern track, without the need to load them from a file. Note that the TSSs automatically used are from the Reference gene model annotations (RefGene) IGB track; the users interested in different annotations, like TSSs or other ones, may choose to load them from another source.

## Results

### Pattern load, search, visualization, comparison, and biological interpretation

The search method implemented in our SimSearch IGB App allows efficiently looking for patterns of (epi)genomic regions within tracks loaded in the Integrated Genome Browser, and visualizing, analyzing, and biologically interpreting the obtained results. It takes advantage of the visual analytics features embedded in IGB and of a set of new additional features that we specifically included in SimSearch.

**Fig. 2:**
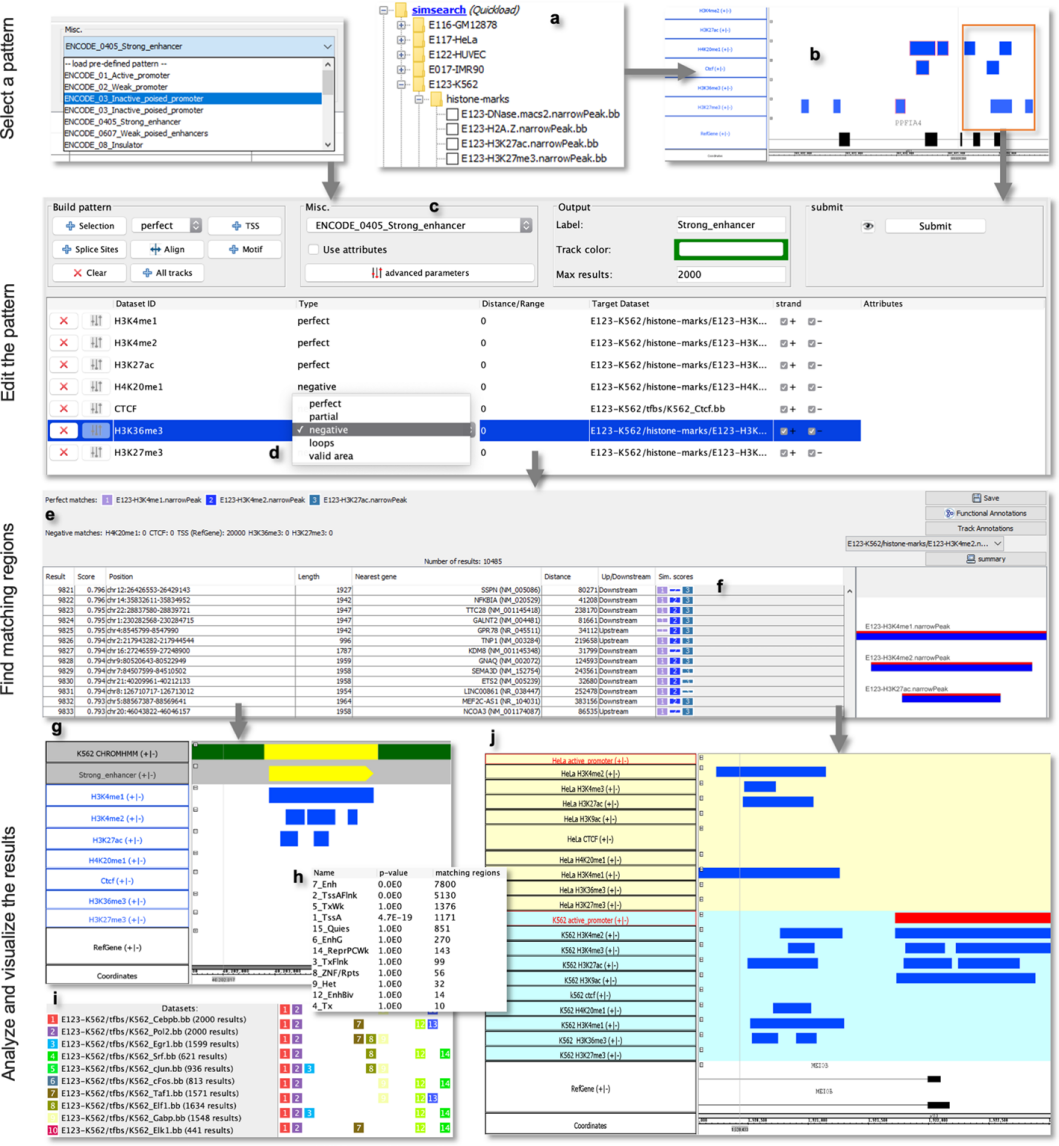
The SimSearch App. *Pattern selection*: Tracks can be loaded in IGB from local or remote files, a DAS server, or a Quickload server (like the one we have prepared so as to provide quick access to peak regions for histone marks and transcription factor bindings in several cell lines (**a**)). A query pattern can be built based on the selection of tracks or track regions (**b**) (with the possibility to select different regions of the same track), or by selecting a pre-defined pattern (**c**). *Pattern editing*: For each track, the type of matching, the relative distance between regions, and the usage of region attributes can be modified; in particular, the type of each pattern track (see Table 1) can be specified (**d**). *Finding matching regions*: The pattern matchings found are displayed in a table (**e**) showing score and features of each identified matching; a visual representation of the pattern matching corresponding to the table row selected is shown on the right of the table (**f**). *Analysis and visualization*: A new track is created (**g**), allowing visual analyses of the results. In this example, the “Strong_enhancer” track, including the result found by SimSearch, shows a region matching the pattern, which overlaps with a similar result from ChromHMM (light yellow: state 7_Enh, Enhancer; dark green: state 5_TxWk, Weak transcription). It is possible to further analyze the results based on annotations from PANTHER [22] or from another IGB track (**h**), or on the composition of the pattern matchings (**i**). **j** Comparison of matchings found for the “Active Promoter” pattern (from ENCODE) in the K562 (light blue background) and HeLa (light yellow background) cell lines. Only in K562 the pattern is found at the TSS of the MEIOB gene, which encodes a chromatin-associated protein required for meiotic recombination and synapsis, in line with the gene expression measured in the two cell lines: 13 FPKM in K562, no expression in HeLa (from the Expression Atlas [23])

After loading (epi)genomic region datasets in IGB tracks, either from files, a DAS server, or a Quickload server (Fig. 2a), the user can define a query pattern “model” based on loaded tracks; it can be a selection of tracks or of specific regions on the tracks (Fig.  2b), for example, peak regions related to histone marks or transcription factors visualized in IGB. In the latter case, the selected regions define the query pattern. By selecting tracks/regions in IGB, the relevant tracks are shown in the SimSearch App panel. For user ease, a list of pre-defined patterns, which we inferred from published results from ChromHMM (see Supplementary Material section S1 for details), is also available from a dropdown menu (Fig. 2c); these patterns characterize several chromatin states, which are defined by the presence (or absence) of histone marks or of the transcriptional repressor CTCF. After selecting any of them, the user can modify/refine it by editing the features of each of its tracks and/or deleting/adding one or more tracks. Target tracks loaded in IGB to be searched for the pattern are automatically matched to the corresponding pattern track based on track name; the user can verify the correctness of the mapping and, in case, modify it.

**Table 1:** Track types and examples

Type	Description	Example
Perfect matching	Matching regions must be present	H3K4me3 histone modification has to be present when looking for the promoter pattern
Partial matching	Regions that are not mandatory but would improve the result if found in the pattern matching	Co-factors
Negative matching	Regions that should not appear in the solution; candidate patterns enclosing such regions are removed from the solution search space	H3K27ac histone modification in enhancer pattern. TSS for distal enhancer pattern. The negative matching track can be associated with a distance (the negative regions are extended on both sides to this distance, e.g., 1,000 bp from TSS)
Valid area	Opposite of negative matching: all regions outside the valid area are removed	TSS neighboring region (e.g., setting a range of 1000 bp around the TSS) in the promoter pattern. Topologically associating domain (TAD) regions
Loop	Interacting regions. Each region is associated with a second region. All regions from other tracks that overlap with one of the two interacting regions are copied to the location of the other interacting region	DNA contact maps inferred from Hi-C experiments: the loops bring physically together a distal enhancer with its associated TSS

Then, the user can decide if the regions in each track describe perfect matchings (default), partial matchings, negative matchings, valid areas, or loops (Fig. 2d). Such choice defines the type of each track, as described in Table 1.

It is also possible to edit a pattern, e.g., to define the expected relative distance between the pattern regions or their length in the case of a pattern defined by track selection, to set the distance required from negative matchings, to specify the attributes of the regions and/or their length to be taken into account when calculating the pattern-matching similarity score, or to automatically add a TSS track from the IGB genome data to the pattern.

Submitting the query pattern makes SimSearch pre-process all target tracks and identify the best pattern matchings they include. The results are shown in a table (Fig. 2e) that displays the chromosome, the coordinates of the centers of the most extreme regions and the overall length of each found matching. For each matching, a graphical representation of its single match in each track is also provided (Fig. 2f); in this, each track is represented as a colored square, whose height is proportional to the single track matching score: a perfect matching with a score of 1.0 is represented by a perfect square, a matching with a score of 0.5 is half its height, while an empty space indicates that no matching was found. This representation allows to understand at a glance which regions are missing in each found matching. SimSearch also associates each identified matching with its nearest gene, defined as the gene with a TSS that is the closest to the center of the root region of the matching (Table 2).

**Table 2:** Annotations of the results provided directly by SimSearch

Type	Description
Nearest gene	For each found matching, the gene with a TSS that is the closest to the center of the root region (i.e., the matching region of the first perfect matching track)
Functional annotations	The PANTHER Web service [22] is used to see if the genes identified in the results (nearest genes) are enriched in particular pathways or biological processes
Annotations from track	The annotations from a track loaded in IGB are used to see if the track regions overlapping with the results are enriched in particular annotations. For instance, it is possible to load the genome segmentation from ChromHMM or Segway to see if the results are associated with particular chromatin states
Sub-patterns	When a search involved several partial matchings, retrieve the tracks whose regions are more often found together

When the user selects a row of the SimSearch result table, a graphical representation of the corresponding pattern matching is displayed on the right-hand side of the table: the regions of the query pattern are displayed in red, the matching regions in blue. This allows a quick understanding of which regions are present or missing in the matching, and how they are aligned to the query pattern regions.

A new track is created in IGB for each executed query; such track displays, for each matching found, a region representing the position and overall length of the matching (Fig. 2g). The user can therefore quickly estimate whether the identified matchings are dense in particular genome areas or spread all over the genome. This type of track also helps comparing the results of different queries, for instance when repeating the search of the same pattern in different samples. Furthermore, the new track(s) can be used for a new search; for instance, it is possible to search first for a distal enhancer pattern, and then use the result to search for transcription factors that bind in the found genomic regions.

SimSearch also includes additional valuable functionalities to ease the functional analysis and biological interpretation of its results (Table 2), besides supporting their evaluation based on the composition of the found pattern matchings (Fig. 2i) (see also Supplementary Material section S3). For instance, it can directly extract genes associated with the result regions (see Supplementary Material section S4) and evaluate if they are enriched for some pathways or biological processes according to available PANTHER annotations [22], or for some annotations in another IGB track (Fig. 2h) (see use case examples below and Supplementary Material sections S5 and S6). The usage of SimSearch is described in detail in its online documentation available at https://deib-geco.github.io/simsearch-app/.

### Use case and evaluation I: genome-wide identification of regulatory regions

The deluge of data generated by large sequencing projects, such as ENCODE or Roadmap Epigenomics, and the development of machine learning algorithms implemented in software like ChromHMM or Segway have made possible to segment and functionally characterize the genome. They allow associating the presence and absence of a selection of histone marks to different chromatin states, such as active promoter regions, enhancers, and many more. In particular, some studies identified several patterns of histone mark combinations as associated with specific chromatin states in some cell lines [7, 13]. Starting from such patterns, with our SimSearch IGB plugin it is possible to easily search efficiently for them, or for similar patterns, genome-wide in different datasets loaded as IGB tracks; this permits, for instance, to infer the regulation state of genomic regions in different cell types or cell lines under different conditions.

To further facilitate this process, we have also pre-compiled several patterns inferred from either the ENCODE or the Roadmap Epigenomics data by ChromHMM (see Supplementary Material section S2). Any of such patterns can be easily selected from a SimSearch dropdown menu and directly used; following the selection, each dataset of the selected pattern is automatically associated with a target track in IGB with the most similar name, thus facilitating the preparation of input data for the search process. The pre-compiled patterns also serve as search templates that can be manually edited for the definition of other new patterns to be used for more refined or broader searches. For instance, by selecting a pre-defined enhancer pattern and adding a TSS dataset as negative matching track with, e.g., a distance of 20,000 bp, it is possible to seamlessly create a new pattern to search for distal enhancers.

As a use case example, in IGB we loaded tracks with histone mark narrow peaks from the Roadmap Epigenomics project for the K562 (human chronic myelogenous leukemia) cell line; then, in SimSearch we selected the pre-defined “strong enhancer” pattern, with the additional condition of having the TSSs at least 20,000 bp away, allowing up to 20,000 results (to speed up the query evaluation, the user can limit the number of results produced by SimSearch; by default, only the 2,000 top-scoring matchings are returned, but the user can change this). Running SimSearch (with default parameters), we obtained 9,117 results, with a matching score over 0.9. To assess their relevance, we annotated them with the chromatin states calculated by ChromHMM on the same dataset, loaded in IGB as an additional track (see Table 2 and Supplementary Material section S6 for details about annotating pattern search results with an IGB track); 7,800 matchings (85% of the total ones) covered 9,892 regions annotated as *Enhancer (Enh)* by ChromHMM (Fig. 2h). Some of these matchings (56% of the total ones) also overlapped with other chromatin states, such as *Flanking active TSS (TssAFlnk) regions, together with the enhancer state region. However, a visual inspection showed that such chromatin states are often flanking the enhancer state regions, thus confirming the result correctness.* Similarly, the results of a search for the “active promoter” pattern resulted enriched for the ChromHMM *Active TSS (TssA)* state (2,000 of the 2,395 matchings found, 83% of them, covered regions annotated as TssA), confirming correctness and relevance of the SimSearch results also in this case.

Chromatin states are different in different cell lines, or under different conditions. To identify and comparatively evaluate them, it is easy to load multiple tracks in IGB (e.g., with datasets from ChIP-seq experiments for the same relevant histone marks under different conditions) and with SimSearch to repeat the pattern search analyses on different sets of them. In this way, it is possible to compare the tracks returned by SimSearch for each search and to identify conserved regulatory regions, or genomic regions differently regulated in different conditions or cell lines (Fig. 2j).

### Use case and evaluation II: identification of transcription factor binding co-occurrences

A number of other important questions in epigenomics and genome regulation concerns the identification of regions where transcription factor (TF) and co-factor bindings co-occur, which TFs bind together, and which are the groups of TFs regulating genes in a particular cell-type or under a particular condition. SimSearch can help provide an answer to such questions, by looking genome-wide for patterns of transcription factors. In IGB we can load, and in SimSearch select, as many tracks as desired concerning the binding sites of a corresponding number of TFs. In addition, it is possible to build a pattern with one or more main TFs of interest (with perfect matching tracks), and many secondary TFs (with partial matching tracks). When searching for similar patterns, SimSearch gives a higher score to the ones containing a higher number of secondary (partial) matchings, while patterns that only match a few secondary TFs are returned with a lower score. The search can be combined with previous results (e.g., enhancer regions identified in previous searches), or with the setting of a distance from the TSSs. Then, in SimSearch the genomic regions found with co-occurrence of TF bindings can be easily annotated with the regions in any IGB track, or they can be directly associated with the nearest gene and functionally annotated with its biological processes and pathways (Table 2) to infer their regulation target.

From the SimSearch output, it is easy to see which TFs have been matched and which have not, thanks to the presence/absence of colored squares in the graphical representation of the SimSearch results (Fig. 2f). Furthermore, one can establish which combinations of TFs are found more often, since a summary visualization shows the number of matchings for each combination of TFs found (Fig. 2i). To facilitate further exploration, it is possible to select a displayed combination in order to automatically create a new search pattern, to be used to search in the same or in a different dataset.

Continuing with the K562 cell line example, we loaded the tracks available in our dataset (see Supplementary Material section S1) for binding sites of transcription factors of the ETS family (ATF3, EGR1, ELF1, ELK1, ETS1, FOS, GABP, JUN, JUND, SP1, SRF, TAF1), which are known to be expressed in hematopoietic cell lines [24]. Following the results of [25], we searched for genomic regions bound by both RNA Polymerase II (Pol2) and CEBPB transcription factors, by loading their tracks and searching for a pattern with both of them set as perfect matching tracks. We reduced the search space to regions marked by DNase-seq (by loading and including their track in the pattern and setting it as valid area track); we added all the ETS family TF tracks as partial matching tracks and allowed for matchings with score $>0.5$ (the minimum score threshold can be set in the advanced parameters configuration window of SimSearch, see *advanced parameters button in* Fig. 2*c)*. We found 2,681 matching genomic regions; the results showed many clusters with up to 3 ETS TFs, including EGR1, JUND and TAF1 that have all been shown to be good indicators of K562-specific activity [25]. Because ETS family members are known to act as monomer, this is likely to illustrate alternative TF bindings at single site.

Similarly, we loaded tracks for other 16 TF binding sites (BCL3, CHD1, EZH2, FOSL1, GATA2, HDAC1, MEF2A, PML, SAP30, SIX5, SP1, SP2, SRF, TAF7, TEAD4, ZBTB33) predicted to be physically associated in several complexes by [26], choosing GATA2 as a root track (perfect matching track) and the other tracks as partial matching, on regions marked by DNase-seq. In the 13,945 results obtained, we found 772 regions matching together FOSL1, GATA2, MEF2A, PML and TEAD4, which [26] had predicted to be a TF complex.

### Not only ChIP-seq

SimSearch is not limited to ChIP-seq datasets, but can be used to search any (epi)genomic region, and its analyses can be extended by integrating multiple other features, e.g., DNase I hypersensitive sites (DHS), transcription start sites, or single nucleotide polymorphisms (SNPs). In the latter case, SimSearch makes possible to identify mutations that may affect TF binding sites or gene regulation in a particular cell line. In addition, we implemented a search on DNA loops that can help identifying additional matchings on track elements brought together by DNA-DNA interaction (see Supplementary Material section S7 and Table 1).

## Conclusions

Large scale bioinformatics analyses are usually followed by expert visual analyses, which are greatly and effectively supported by visual analytics tools. As it is well known to researchers, a powerful search algorithm is more useful when combined with an effective visualization tool [27], allowing them to visually assess the biological meaning of the search matchings found, e.g., by analyzing the signal peaks in the result regions, looking at neighboring genes, or comparing the results with each other or with additional data.

Out of the many genome browsers available, besides supporting some visual analytics task, IGB offers the advantage of being easily extendable with plugins. This strategy offers several advantages:

1. it allows relying on the features of IGB for the main tasks (genomes and data loading, interaction and zooming, etc.), and benefiting from all the future developments of IGB;
2. it can display both online data (without the need for the user to perform space and time consuming downloads of genomes and datasets) and local data (to speed up the analyses or to ensure the privacy of the data);
3. it permits the integration of all the analyses and visualizations in a single application, without forcing the user to install several independent tools and to switch between them to perform the requested analysis.

We developed an efficient pattern-search algorithm and included it in the easy-to-use SimSearch IGB plugin, extending the IGB visual analytics capabilities to provide new functionalities for (epi)genomics. SimSearch provides scientists with the ability, once they identify (possibly directly on the genome browser) an interesting (epi)genomic region pattern, to seamlessly look for similar occurrences of such pattern in the whole genome, and directly analyze the obtained results.

Our work well complements previously proposed approaches, like ChromHMM and Segway. They require to process aligned read data to search for patterns of interest, which is a time-consuming task; conversely, our approach applies pre-defined patterns, e.g., those found by ChromHMM or Segway, directly on processed data, so as to quickly and effectively search for their occurrences genome-wide.

We reported the results obtained in the evaluation of the several functionalities of SimSearch; they are in accordance with those already known in the literature; this demonstrates the efficacy and relevance of our method and the high usefulness of SimSearch in support of the search, discovery, identification, and biological interpretation of interesting findings in the whole (epi)genomic context.

## Supplementary information

**Additional file 1:** Supplementary material describing: **S1** Quickload server, **S2** Patterns inferred from ChromHMM, **S3** Sub-patterns found, **S4** Nearest gene annotation, **S5** Functional annotations, **S6** Annotation from tracks, **S7** DNA loops, and related References.

## Data Availability

SimSearch IGB plugin, documentation and code are freely available at https://deib-geco.github.io/simsearch-app/ and https://github.com/DEIB-GECO/simsearch-app/.

## Abbreviations

BED: Browser extensible data
bigBed: big BED format
bigWig: big Wiggle format
DAS: Distributed annotation system
DHS: DNase I hypersensitive sites
DNA: Deoxyribo nucleic acid
ENCODE: Encyclopedia of DNA elements
Enh: Enhancer
GeCo: Data-driven genomic computing
GTF: Gene transfer format
IGB: Integrated genome browser
IGV: Integrative genomics viewer
NGS: Next generation sequencing
SNP: Single nucleotide polymorphism
TAD: Topologically associating domain
TF: Transcription factor
TssA: Active TSS
TssAFlnk: Flanking active TSS
VCF: Variant call format

## Availability and requirement

Project name: SimSearch. Project home page: https://deib-geco.github.io/simsearch-app/. Project source code and issue tracker: https://github.com/DEIB-GECO/simsearch-app/. Operating system(s): Platform independent. Programming language: Java 8. Other requirements: Integrated Genome Browser. License: The SimSearch IGB plugin is released under the Apache License 2.0; the pattern-search algorithm is copyrighted by the University of Bologna. Any restrictions to use by non-academics: The pattern-search algorithm is free for non-commercial use only

## References

[1] Goodwin S (2016). Coming of age: ten years of next-generation sequencing technologies. Nat. Rev. Genet..

[2] ENCODE Project Consortium (2012). An integrated encyclopedia of DNA elements in the human genome. Nature.

[3] Bernstein BE (2010). The NIH roadmap epigenomics mapping consortium. Nat. Biotechnol..

[4] Ernst J, Kellis M (2010). Discovery and characterization of chromatin states for systematic annotation of the human genome. Nat. Biotechnol..

[5] Ernst J (2011). Mapping and analysis of chromatin state dynamics in nine human cell types. Nature.

[6] Roy S (2010). Identification of functional elements and regulatory circuits by Drosophila modENCODE. Science.

[7] Ernst J, Kellis M (2012). ChromHMM: automating chromatin state discovery and characterization. Nat. Methods..

[8] Baum LE, Petrie T (1966). Statistical inference for probabilistic functions of finite state Markov chains. Ann. Math. Stat..

[9] Hoffman MM (2012). Unsupervised pattern discovery in human chromatin structure through genomic segmentation. Nat. Methods.

[10] Chan RCW (2018). Segway 2.0: Gaussian mixture models and minibatch training. Bioinformatics.

[11] Dagum, P. et al.: Dynamic network models for forecasting. In: Proceedings of the Twenty-Eighth International Joint Conference on Artificial Intelligence. AUAI Press, pp. 41–48 (1992).

[12] Bilmes J.: Dynamic Bayesian multinets. In: Boutilier, C., Goldszmidt, M. (eds) UAI ’00: Proceedings of the 16th Conference on Uncertainty in Artificial Intelligence, Morgan Kaufmann, San Francisco, CA, pp. 38–45 (2000).

[13] Roadmap Epigenomics Consortium (2015). Integrative analysis of 111 reference human epigenomes. Nature.

[14] Kuhn RM (2013). The UCSC genome browser and associated tools. Brief. Bioinform..

[15] Skinner ME (2009). JBrowse: a next-generation genome browser. Genome Res..

[16] Thorvaldsdóttir H (2013). Integrative Genomics Viewer (IGV): high-performance genomics data visualization and exploration. Brief. Bioinform..

[17] Nicol JW (2009). The Integrated Genome Browser: free software for distribution and exploration of genome-scale datasets. Bioinformatics.

[18] Freese NH (2016). Integrated genome browser: visual analytics platform for genomics. Bioinformatics.

[19] Montanari P (2016). Pattern similarity search in genomic sequences. IEEE Trans. Know. Data Eng..

[20] Helt GA (2009). Genoviz Software Development Kit: Java tool kit for building genomics visualization applications. BMC Bioinform..

[21] Rao SS (2014). A 3D map of the human genome at kilobase resolution reveals principles of chromatin looping. Cell.

[22] Mi H (2013). Large-scale gene function analysis with the PANTHER classification system. Nat. Protocol..

[23] Petryszak R (2016). Expression Atlas update: an integrated database of gene and protein expression in humans, animals and plants. Nucleic Acids Res..

[24] Clausen PA (1997). ETS-1 induces increased expression of erythroid markers in the pluripotent erythroleukemic cell lines K562 and HEL. Leukemia.

[25] Savic D (2015). Promoter-distal RNA polymerase II binding discriminates active from inactive CCAAT/ enhancer-binding protein beta binding sites. Genome Res..

[26] Giannopoulou EG, Elemento O (2013). Inferring chromatin-bound protein complexes from genome-wide binding assays. Genome Res..

[27] Schroeder MP (2013). Visualizing multidimensional cancer genomics data. Genome Med..

