# Supplementary material for: Search and comparison of (epi)genomic feature patterns in multiple genome browser tracks
Source: BMC Bioinformatics. 2020 Oct 19;21:464. doi: 10.1186/s12859-020-03781-2 (PMC7574191; doi:10.1186/s12859-020-03781-2)
Supplement: Supplementary file 1 — Additional file 1: Supplementary material describing: S1 Quickload server, S2 Patterns inferred from ChromHMM, S3 Sub-patterns found, S4 Nearest gene annotation, S5 Functional annotations, S6 Annotation from tracks, S7 DNA loops, and related References. [file 12859_2020_3781_MOESM1_ESM.pdf]

Supplementary Material

S1 Quickload server

To facilitate the testing and usage of our pattern search method and of its SimSearch implementation, we provide several datasets of epigenomic features for six human cell lines: GM12878, HeLa, HUVEC, IMR90, K562 and NHEK. For each cell line, we downloaded the datasets of transcription factor binding sites, histone modification sites, and DNase I hypersensitive sites (DHS) from the ENCODE and Roadmap Epigenomics projects, by using the UCSC Goldenpath server (http://hgdownload.cse.ucsc.edu/goldenpath/hg19/encodeDCC/wgEncodeOpenChromChip/) and the NIH Roadmap Epigenomics Web portal (http://egg2.wustl.edu/roadmap/data/byFileType/peaks/consolidated/), respectively.

We completed all datasets with their chromatin state annotations from ChromHMM (Ernst and Kellis, 2012 - http://egg2.wustl.edu/roadmap/data/byFileType/chromhmmSegmentations/ChmmModels/coreMarks/jointModel/final/) and Segway (Libbrecht *et al.* , 2019 - http://noble.gs.washington.edu/proj/encyclopedia/). All data files were converted to bigBed format with the bedToBigBed application (Kent *et al*., 2010).

We also downloaded the contact maps from (Rao *et al*., 2014) available from GEO (Clough and Barrett, 2016) (GEO accession: GSE63525, ftp://ftp.ncbi.nlm.nih.gov/geo/series/GSE63nnn/GSE63525/suppl/), which we converted to BED format.

All the datasets were downloaded for the genome assembly hg19. In addition, we converted the genomics coordinates of histone marks and transcription factor binding sites for hg38 with Liftover (https://genome.ucsc.edu/cgi-bin/hgLiftOver).

Access to all these data is provided by a Quickload server (Freese *et al*., 2016), which we created and made available at https://github.com/DEIB-GECO/simsearch-quickload/. The scripts used for downloading and processing the source data into the Quickload server are released in a GitHub public repository (https://github.com/DEIB-GECO/simsearch-quickload/).

S2 Patterns inferred from ChromHMM

We used the results of two published studies to define two lists of patterns reflecting a representative panel of histone marks. Both studies used ChromHMM to predict chromatin states from the results of ChIP-seq, DNAse-seq, and FAIR-seq next generation sequencing experiments. The first study (Ernst and Kellis, 2012) used data from the ENCODE project for 8 histone marks (H3K4me1, H3K4me2, H3K4me3, H3K9ac, H3K20me1, H3K27ac, H3K27me3, H3K36me3) and for CTCF binding sites, and inferred 15 chromatin states (see Figure 1b of Ernst and Kellis, 2012). In the second study (Roadmap Epigenomics Consortium, 2015), the Roadmap Epigenomics Consortium reports 15 chromatin states predicted by ChromHMM based on analyses of the ChIP-seq experiments on 5 histone marks (H3K4me1, H3K4me3, H3K9me3, H3K27me3, H3K36me3, see Figure 4a and 4b of the article, available at http://egg2.wustl.edu/roadmap/figures/mainFigs/Figure_4.jpg).

For 21 chromatin states reported in the two studies, we inferred the histone mark and CTCF patterns associated with each of them by visually inspecting the heatmaps provided in the two articles. In every pattern, we set each histone mark or CTCF dataset as a perfect matching or negative matching track (see Table 1 in the main paper) according to the intensity of its signal in the heatmap; datasets with medium intensity were added as partial matching tracks, reflecting the idea that they are desired, but not required. Finally, we merged the patterns from the same study regarding the same chromatin state, like states 4 and 5 (Strong enhancer), or 6 and 7 (Weak/poised enhancer) in the first study (for the annotation of the chromatin states see also https://genome.ucsc.edu/cgi-bin/hgTrackUi?g=wgEncodeBroadHmm&db=hg19).

S3 Sub-patterns found

For each pattern search performed using SimSearch, all combinations of datasets (perfect and partial matchings) are calculated and assigned to the number of times each combination has been found across the genome (see Figure 2i in the main paper). This can help the user identifying groups of datasets (for instance, transcription factors) that often bind together. On request, counts can be displayed as a list. To improve clarity of the visualization, smaller combinations are only displayed if they are found more often than complex ones (for instance, if 100 instances of A + B + C and 100 instances of A + B are found, only A + B + C is displayed).

S4 Nearest gene annotation

Each pattern matching that SimSearch finds is associated with the nearest gene, defined as the one with a transcription start site (TSS) that is the closest to the center of the root region of the pattern matching. If the TSS is in a track defined of type “perfect matching” for the pattern, it is selected as root region; in any other case, the root region is in the first perfect matching track of the pattern.

S5 Functional annotations

To support assessing the biological function of the genomic regions found as pattern matchings, SimSearch automatically submits the list of their nearest genes (a gene for each region) to the PANTHER classification system Web service (Mi *et al*., 2013), repeating the query separately for biological processes and for pathways. For each annotation returned by the PANTHER Web service, SimSearch displays the associated p-value [with Bonferroni's correction (Bonferroni, 1936; Dunn, 1961)], the number of genes associated, and the names of such genes. No filter is applied on the p-value; so, all annotations are displayed.

S6 Annotation from tracks

An Integrated Genome Browser (IGB) (Nicol *et al*., 2009; Freese *et al*., 2016) track (not necessarily from those used for pattern searching) can be used to annotate the pattern search results with SimSearch. In this case, the genomic regions from such a track, classified according to their name (e.g., a chromatin state), are associated with the pattern matchings found overlapping with them. SimSearch counts the number of all regions of each class in the track that overlap with the pattern matchings found, as well as the number of all regions of each class in the track that do not overlap, and it performs a Fisher exact test (Fisher, 1922) to assess the significance of the association of the pattern matchings with each annotation class (contingency table rows: selected annotation and other annotations, columns: annotations covered by a matching and annotations not covered).

S7 DNA loops

The SimSearch plugin also supports the particular case of chromosome conformation capture (Grob and Cavalli, 2018) datasets (such as contact maps from Hi-C experiments). In the absence of a standard for the description of contact maps, we used a BED6 format, where the blocks defined in columns 10 to 11 represent two interacting regions. SimSearch stores the coordinates of each pair of interacting regions; when other tracks are imported, each of their regions that falls into a contact region is copied to the location of the related interacting region. This allows identifying pattern matchings formed by distant regions brought closer by long-range chromosome interactions.

In the main paper, we have shown how SimSearch can efficiently find similar patterns in multiple different datasets, which are often associated with regulatory regions. In the use cases illustrated in the main paper, results are mapped on single regions of the genome, providing a neat although simplified view of genome regulation. Yet, genome regulation is broader, with genes indeed regulated not only by promoters and neighboring enhancers, but also by distal elements. Contacts in the 3D space between proximal and distal elements define regulatory long-range chromosome interactions, which can be identified, e.g., by chromosome conformation capture assays such as Hi-C experiments. Processing of Hi-C experiments generates two types of data: topologically associating domains (TADs) and contact loops. TADs are genomic regions where DNA contacts occur frequently. Within TADs, loop ends are the regions, within or between chromosomes, that are in physical contact.

Moving toward the integration of 3D genome interactions into a genome browser, in SimSearch we defined two related types of tracks: “valid area” and “loop” (Table 1 in the main paper). The first one should be assigned to tracks including regions of interest. When associated with TADs, SimSearch only finds matchings within those TADs. Furthermore, we added the possibility to import and seamlessly use DNA contact information (DNA loops). When the user chooses to assign the loop type to a track, SimSearch simulates the presence of the regions of the other tracks on both sides of a DNA loop (Figure S1). With this feature, for instance, in a new search we can combine the resulting track of the “distal strong enhancer” search presented in section 3.2 of the main paper with TSS sites (both as perfect matching), by including loop regions from Rao *et al*., 2014; the results suggest targets for the enhancers previously detected (Figure S1c). To further assess the relevance of these advanced options, we extended the pattern regarding RNA Polymerase II (Pol2) and CEBPB transcription factors, considered in the second use case presented in the main paper, in section 3.3, by including the same loop regions from Rao *et al*., 2014. We found 2,750 results (2,681 without considering loop regions). Visual inspection of the results shown that they associate Pol2 and CEBPB with distal regions that could come in contact thanks to chromosome loops, and thus influence their regulatory activity, as well as with additional distant transcription factors.

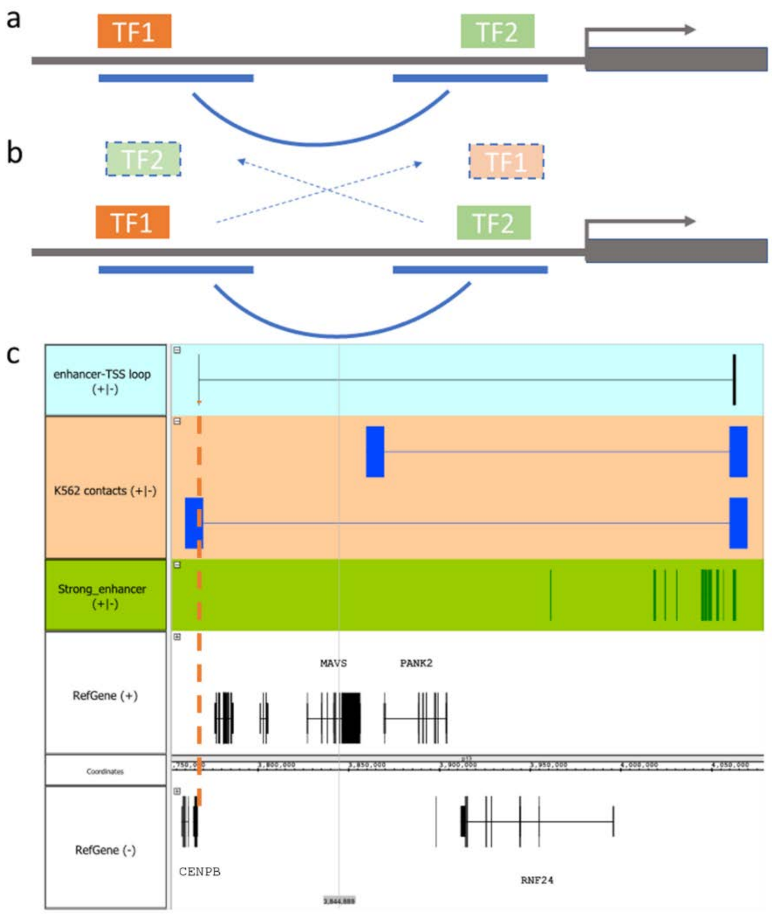

Fig. S1. Pattern matching in DNA loops. **(a)** Two transcription factors TF1 and TF2 bind distant genome regions that are part of a DNA loop (blue line). **(b)** SimSearch projects the binding regions on both side of a DNA loop and, in the example, suggests a new pattern matching region close to the TSS of a target gene. **(c)** Example of DNA loop pattern: searching for distal strong enhancer (resulting track of previous query, green background) matching a TSS through DNA-loop contacts (orange background track) in the K562 cell line. Result track (light blue background): a distal enhancer [right on the green background track, corresponding with the enhancer site GH20J004081 from the GeneHancer database (Fishilevich *et al*., 2017)] matches the TSS of the CENPB gene [left on the reverse strand (RefGene (-) track), as highlighted by the vertical orange dashed line] distant 295,000 bp, thanks to the longer DNA-loop contact shown in the orange background track.

References

Bonferroni,C.E. (1936) Teoria statistica delle classi e calcolo delle probabilità. *Pubblicazioni del R Istituto Superiore di Scienze Economiche e Commerciali di Firenze*, **8**, 3-62.
Clough,E. and Barrett,T. (2016) The Gene Expression Omnibus Database. *Methods Mol. Biol*., **1418**, 93-110.
Dunn,O.J. (1961) Multiple comparison among means. *J. Am. Stat. Assoc*., **56**, 52-64.
Ernst,J. and Kellis,M. (2012) ChromHMM: automating chromatin state discovery and characterization. *Nat. Methods*., **9**(3), 215-216.
Fisher,R.A. (1922) On the interpretation of X2 from contingency tables, and the calculation of P. *J. R. Stat. Soc*., **85**(1), 87-94.
Fishilevich,S. *et al*. (2017) GeneHancer: genome-wide integration of enhancers and target genes in GeneCards. *Database*, **2017**, 2017.
Freese,N.H. *et al*. (2016) Integrated genome browser: visual analytics platform for genomics. *Bioinformatics*, **32**(14), 2089-2095.
Grob,S. and Cavalli,G. (2018) Technical Review: A hitchhiker's guide to chromosome conformation capture. *Methods Mol. Biol*., **1675**, 233-246.
Kent,W.J. *et al*. (2010) BigWig and BigBed: enabling browsing of large distributed datasets. *Bioinformatics*, **26**(17), 2204-2207.
Libbrecht,M.W. *et al*. (2019) A unified encyclopedia of human functional elements through fully automated annotation of 164 human cell types. *Genome Biol*., **20**(1), 180.
Mi,H. *et al*. (2013) Large-scale gene function analysis with the PANTHER classification system. *Nat. Protoc*., **8**(8), 1551-1566.
Nicol,J.W. *et al*. (2009) The Integrated Genome Browser: free software for distribution and exploration of genome-scale datasets. *Bioinformatics*, **25**(20), 2730-2731.
Rao,S.S. *et al*. (2014) A 3D map of the human genome at kilobase resolution reveals principles of chromatin looping. *Cell*, **159**(7), 1665-1680.
Roadmap Epigenomics Consortium. (2015) Integrative analysis of 111 reference human epigenomes. *Nature*, **518**(7539), 317-330.
